# “We Can All Learn Together so We’re All on the Same Page”: Impact of a Learning Essential Approaches to Palliative Care Hospital Course on Hospitalists’ Practice

**DOI:** 10.1089/pmr.2024.0094

**Published:** 2025-05-05

**Authors:** Ashlinder Gill, Sarah Romeril, Lynn Meadows, Alison Flanagan, Ashwak Rhayel, Michael Panza, Christopher Klinger, Narisa Duboff, Jose Pereira, Joan Bellaire

**Affiliations:** ^1^ Division of Palliative Care, Department of Family Medicine, McMaster University, Hamilton, Canada.; ^2^ School of Nursing, McMaster University, Hamilton, Canada.; ^3^ Department of Community Health Sciences, University of Calgary, Calgary, Canada.; ^4^ University of Toronto, Toronto, Canada.; ^5^ Educación Médica y Cuidados Paliativos, Facultad de Medicina y Instituto Cultura y Sociedad (ICS). Universidad de Navarra, España, Spain.; ^6^ Scientific Advisor, Pallium Canada, Ottawa, Canada.; ^7^ Family Medicine, Hamilton Health Sciences, Grimsby, Canada.

**Keywords:** hospitalist, medical education, palliative care

## Abstract

**Background:**

In Canada, access to palliative care varies across jurisdictions. Many health care professionals lack core palliative care competencies. To help build capacities, a pilot education program was conducted at a community hospital in Southwestern Ontario (Canada). Using Pallium Canada’s Learning Essential Approaches to Palliative Care (LEAP) Hospital course, generalist hospital physicians participated in this initiative. The purpose of this investigation was to explore the impact of the LEAP Hospital course on physician attitudes, comfort, and care delivery.

**Methods:**

Within a Plan-Do-Study-Act framework, a mixed-methods design summarized post-course evaluations, commitment to change (CTC) statements, and interviews with learners and hospital staff. Participants completed pre-course knowledge and post-participation surveys. Group and individual interviews were conducted with learners and staff who practiced alongside LEAP learners. Descriptive statistics were completed for aggregate survey data. Thematic analysis was conducted to summarize learner and staff experiences.

**Results:**

Twenty-nine physicians completed the LEAP Hospital course. Ninety-six CTC statements identified improvements in symptom management and communication. Sixteen participants participated in interviews. Learners and hospital staff noted the impact courses had on symptom and disease management and enhanced communication when discussing goals of care. Learners valued case-based learning and connecting with peers who are often siloed in practice. Participants also noted the inclusion of allied health for greater collaboration.

**Conclusions:**

LEAP Hospital courses enhanced knowledge and skills and incorporated a greater palliative approach to care. Establishing a community of practice to address educational needs and strategies should be considered while supporting the inclusion of new graduates and hires.

## Introduction

Numerous studies have highlighted significant needs of patients with advanced cancer and noncancer illnesses.^1–4^ While managing advanced disease, delays with advance care planning and goals-of-care discussions, inappropriate treatments, and misuse of health care resources have all led to patients having unmet needs.^5,6^ Despite improvements in the last decade, many patients across Canada and internationally who require palliative care are still not getting access to it.^7,8^ Several factors account for this, including the lack of specialist-level palliative care clinicians and services and gaps in palliative care literacy across the health professions.^9^

In Canada, access to palliative care services resembles a patchwork, where variability across jurisdictions and care settings exists, ranging from palliative care specialist teams or health care professionals with core palliative care competencies working in primary care, and other specialties. In Ontario, some hospitals have palliative care teams and others have health care professionals who have acquired core palliative care skills, while others have minimal or absent palliative care teams and/or staff with core competencies in palliative care.^10^ Gaps in care delivery include the absence of, or inadequately resourced hospital or community palliative care consultation teams, inadequate numbers of palliative care specialist physicians and nurses, and suboptimal numbers of palliative care unit beds and residential hospice beds.^11–13^

Many health care professionals lack core competencies of palliative care or need their skills updated, including hospital-based clinicians and staff hospitalists.^14–17^ Core competencies can be acquired during undergraduate and postgraduate education and by continuing professional development for those already in practice.^10^ In Canada and internationally, growing opportunities exist for palliative care education to develop primary-level palliative care across different settings and professions.

To build capacity among the clinicians and staff to provide a palliative approach to care, an education program was undertaken using Pallium Canada’s Learning Essential Approaches to Palliative Care (LEAP) Hospital course.^11^ Courses use small-group, case-based learning, and are designed for different care settings and disease groups.^10^ LEAP Hospital is an interprofessional course that provides health care professionals with essential skills and competencies of a palliative care approach, with course modules and case studies contextualized to the hospital setting.

This is the first investigation to assess the impact of the LEAP Hospital course as a part of a broader pilot education-based quality improvement program for a community-based hospital. The primary objective of this investigation was to explore the impact the LEAP Hospital course had on the knowledge, attitudes, commitments to change, and clinical practice of those who took the course, as experienced by the participants themselves and colleagues who work with them.

## Methods

### Study design

A mixed methods approach was used with the collection of qualitative and quantitative data,^18^ within a PDSA cycle^12^ of this pilot education-based quality improvement project. The *do* phase was LEAP hospital training, which was conducted virtually with a cohort of generalist hospital physicians. As a part of the study phase, data were sourced using four techniques: (a) Pallium Canada’s^13^ learner course evaluations and commitment-to-change (CTC) statements, and (b) focus group and individual interviews with LEAP learners, and (c) interviews with hospital staff who interacted with LEAP learners. The study was approved by the Hamilton Integrated Research Ethics Board (HiREB #15478). A sequential explanatory mixed methods design^14^ was applied to this investigation. Survey-based data completed by course participants were reviewed to observe a change in participant attitudes and comfort with a palliative approach to care. Learners were then invited to participate in a semi-structured interview about the impact of course teaching on their practice. Staff who worked with learners were also invited to participate to better understand the impact of learning on care delivery and staff collaboration. Both survey and interview data were connected during analysis and interpretation within the “study” phase to better understand course impact on practice and care delivery.

### Setting and intervention

LEAP Hospital training was conducted remotely for hospital staff in a rural area of Southwestern Ontario (Canada).

The community hospital is part of a larger health care system providing both inpatient and outpatient services, including palliative care. The hospital is an associated teaching site for McMaster University, and physician staffing includes hospitalists and local (family) physicians with hospital privileges. This site comprises 50 beds, including an active emergency department, complex continuing care, and one dedicated palliative bed. The palliative care team is population-based and covers multiple settings of care across the local region: in hospital, hospice, long-term care, and the community. The team consists of 0.6 FTE physician funded through an Alternate Funding Plan, 2 part-time fee-for-service physicians, 1.0 clinical nurse specialist, 0.8 program secretary, 0.5 psychosocial support, and 0.5 bereavement counseling. In the past year, the physician/nurse team conducted a total of 823 consultations; 61% were inpatient visits, and 39% were community visits (home and hospice care).

While the original goal was to deliver the classroom, one-day version of the LEAP Hospital course, the project had to switch to the fully online version of the course because of social restrictions imposed by the COVID-19 pandemic. The online version consists of two parts: Part 1 is a series of 15 self-learning, online modules, followed by Part 2, four facilitated live webinars (each 90 minutes long). The webinars use case-based learning in which theory covered in the self-learning modules is applied through cases. Two courses (or sessions)—both in early 2022—were delivered to accommodate all the learners, as the maximum number of learners per course is limited to 20 to 25 to ensure interactivity. The courses were facilitated by members of the hospital and regional palliative care team, who are trained and certified LEAP facilitators. This facilitated one of the goals of the project, to increase collaboration between the palliative care team and hospital clinicians.

### Data collection

All LEAP courses and learners are registered online using Pallium’s Learning Management System platform for submitting course-related surveys and questionnaires, including pre- and post-course questionnaires and CTC statements. Pre-course, learners completed three online standardized LEAP instruments that explore different aspects of providing a palliative care approach: a Knowledge Quiz, a self-Perceived Comfort Scale, and an Attitudes Scale. These instruments were developed specifically for the purposes of the LEAP courses and validated. The Knowledge Quiz is a 20-item multiple-choice questionnaire.^13^ The Comfort Scale (16 items) and the Attitudes Scales (20 items) both use Likert-type scales. Post-course, learners completed the same instruments again, as well as a standardized course evaluation survey. Although these learning tools prompt reflection, they are also used to evaluate the impact of the courses.^15^ Post-course, learners are also asked to identify three to four things that they will do differently because of course participation. This CTC approach has been shown to increase implementation following an education intervention.^16,17^ Four months after completing the course, each learner who submitted CTC statements was automatically sent a personalized email to reflect on their initial CTC statements and to indicate whether they implemented them.

Focus group and individual interviews were conducted with physicians who participated in the LEAP Hospital courses, and health care providers who work along with course participants to better understand course impact on physician practice and broader care delivery. Purposeful sampling^19^ was conducted to ensure LEAP learners and staff were contacted for participation. Recruitment occurred via email invitation. The study’s heterogeneous participants, narrow research question(s), study design, and semi-structured interview guides suggested a medium size sample (approximately 10 participants) would help describe experiences related to the impact of LEAP hospital training.^17^ Staff participated in either in-person or virtual interviews, depending on participants’ availability and preference. Written informed consent was conducted prior to the interview, and verbal consent for audio recording. Researchers facilitated interviews using a semi-structured interview guide, and explored participant background, clinical duties, LEAP Hospital participation (for learner participants only), and its impact on care provision. Interviews were audio-recorded and transcribed verbatim.

### Data analyses

Quantitative data, consisting of pre- and post-course surveys, were analyzed descriptively in aggregate form. Descriptive statistics included means, medians, ranges, and standard deviations of survey responses using Microsoft Excel™. The small number of participants did not allow for inferential analyses. Thematic content analysis^18^ was applied to identify recurring ideas or topics specific to course participation and its impact on care delivery. Transcripts were uploaded to NVivo 2020™ for data management and coding. Analysis identified recurring topics, which became more refined as the data were being collected and compared. Research team members independently coded each transcript, before iteratively comparing topics that were then further analyzed to identify consensus the identified themes. To ensure rigour, coders adopted triangulation of data, analysis of data by multiple team members, and consensus discussions with interdisciplinary team members.^20^ Bracketing, reflexivity, peer review, and an audit trail further supported rigour.^20^

## Results

Data from all sources demonstrated the impact of course teaching on staff beliefs, care delivery, and their knowledge and attitudes towards palliative care.

### A. LEAP course evaluations

Twenty-nine physicians completed the online LEAP Hospital course modules and attended a single-session virtual webinar across two sessions. Learners were exclusively physicians as they received CME credits for the course and fees were waived. Other health care providers do not have similar accreditation and would not have had the fees waived. Most participants were family physicians, and self-identified as female. All physicians had hospital privileges and considered the most responsible physician for their own patients. Seven of 29 learners later participated in qualitative interviews. Learners who did not complete interviews and/or evaluations were due to nonresponse. Twenty-four learners (82%) completed the LEAP pre-course, and 14 (48%) completed the post-course knowledge quiz. The average scores increased from 11 points pre-course to 17 post-course. (Supplementary Appendix A1) The LEAP Hospital course was viewed favorably, meeting learning needs by participants and rated highly for relevancy to practice. (Supplementary Appendix A2).

Course evaluations also included three open-text questions, about (a) course improvements (b) suggestions for additional content, and (c) what worked best on the course. Responses included learner preferences for in-person learning, interactions, and discussion, as opposed to online didactic learning. Learners’ feedback on overall course logistics included suggestions for course organization, and increased time on cases and group discussions. Other suggestions included changing the module order to focus on medicine-heavy topics earlier and providing more complex scenarios addressing inpatient palliative care. Suggestions for future course content included addressing active treatment earlier in the patient’s trajectory, MAID, sharing bad news, and writing pump orders. Some participants appreciated the online format, highlighting the convenience and ability to go at one’s own pace as beneficial.

### b. Commitments to change statements

CTC statements were submitted by 22 learners (75%) and 4-months commitment reflections by seven participants (31%), resulting in a total of 96 statements for review. The most common topics identified from CTC statements were *symptom management, incorporating a more palliative approach to care, advance care planning,* and *having the appropriate language and communication to facilitate complex discussions.* (Table 1) *Symptom management* CTCs reflected optimal treatment strategies and first-line options for managing complex symptoms, having the knowledge to become more confident and independent in their administration during care planning. *Incorporating a palliative approach to care* included upstream discussions of advanced care planning, early identification of patients needing such discussions (e.g., both cancer and chronic disease management), using the appropriate resources for this (e.g., applying the surprise question for patient identification), and engaging in goals-of-care discussions overall were listed.

**Table 1.** Commitments to Change: Themes and Frequencies

Commitment to change statements	Frequency (total statements *n* = 96)	Description and examples
Symptom Management	29**	Improve symptom management. *“More aggressive symptom control in last days and hours.”*
Palliative Care Approach	24	Initiate palliative care earlier in the illness trajectory and across cancer and noncancer illnesses. *“Recognize/pay more attention to when patients may be entering that transition phase between active/acute care and palliative care”*
Advance Care Planning	19	Improve advance care planning and initiate it more regularly. *“Start discussing ACP with all of my patients when appropriate.”*
Symptom Screening	16	Undertake more frequent and standardized screening for symptoms and needs in daily practice. *“I will assess for symptoms using the ESAS more regularly in my practice.”*
Communication and patient centeredness	15	Improve communication approaches and more patient centeredness. *“Repeatedly asking about personal values and goals when talking about a patient’s management plan, and using the “I wish… I worry... I wonder” approach to help approach transitions in functional status and decision-making.”*

**A single statement can be coded across multiple categories.

### (c) Interview data

Sixteen participants provided additional insights into the impact of LEAP Hospital training on their professional practice. Of these participants, seven were LEAP learners and nine were hospital staff members who worked alongside LEAP learners (but did not participate in the course themselves). Most LEAP learners had greater than 10 years of clinical experience, provided locum hospitalist support, and conducted visits at home and/or hospice. Hospital staff who provided additional context to LEAP training included palliative care physicians, discharge planning, nursing, psychosocial counseling, rehabilitation and dietary staff. To protect anonymity, participants have been labeled as palliative care physician or allied health.

**FIG. 1.**
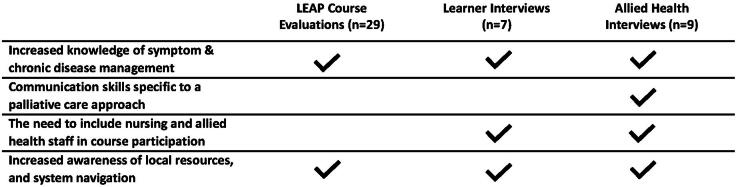
Illustration of how identified topics describing the impact of LEAP Hospital emerged from multiple data sources.

After reviewing interview data, identified topics highlighted the specific impact course teachings had on symptom and advanced disease management and providing opportunities for greater interprofessional collaboration and networking. Learners provided additional suggestions and improvements for future learnings, such as including more allied health providers (such as hospital and community nursing) and incorporating material specific to local resources, including available resources for patients who require additional support at home. (Table 2) A summary of course feedback (from post-course evaluations and CTC statements) and identified themes on course impact were compared across data sources to further demonstrate triangulation of identified topics across the multiple data sources and facilitate integration of findings. (Fig. 1).

**Table 2.** Identified Themes Describing the Impact of LEAP Hospital Course from Interview Data

Theme	Coding frequency (number of references coded across number of interviews)	Description	Example quote
Symptom Management	13 references across 6 interviews	“Refresh” of newer therapies and treatments Referrals more upstream, including chronic disease management Ability to have more “difficult” discussions	“*Yeah, it was very helpful to: one, bring it to the forefront that, you know, palliative care is not just for those that are like, more acutely dying or dying in the short-term, but those that might, you know, have chronic disease that will have, you know, kind of like a near-death experience over time, maybe multiple times*.” [Learner A] , “*… I did notice before* [electronic medical record] *came in, like I started in May and [EMR] came in in June, and I noticed more [haloperidol] use in this region for nausea than in other regions previously. Like, usually in acute care hospitals it’s [dimenhydrinate] or ondansetron, and it’s an uphill battle all the way...”* [Palliative care Physician A] “*So, I would speak to like what I saw right away after we did it, so I would say one of the biggest things was early and more appropriate consults. So, initially when before we started the project we were getting a lot of referrals, which were great at our like very low PPS, like 10’s, 20’s, and I think I feel like subjectively I noticed a big difference in that there was like more early upstream referrals that were higher PPS and higher functional status, so we felt like we could have more of an impact on them. And then a lot more for medical conditions that were not necessarily like obvious cases like malignancy”. …”* [Palliative care physician A] *So, we saw a lot more, a lot of referrals actually from long-term care, for cognition, memory, dementia and generally speaking more CHF…”* [Palliative care physician A] “*I think they are having maybe more blunt conversations. And even with, I think our code status is with family members too, they do revisit sometimes, and they’re giving more conversations about what actually, you know, CPR entails. … So they’re having, I think, a little more in depth conversations with people.”* [Allied Health 1]
Collaborative Care	13 references across 7 interviews	Learning with colleagues increased collaboration and networking. Increased application of the palliative care approach. Identified need for interprofessional palliative care training in future.	“*And I guess that’s another thing I can comment on, is that I remember that I think most of the course attendees were people that I knew and worked with clinically. And I think it’s always really helpful—like at* [hospital]*, especially, we’re a fairly small facility and so we work together quite closely as a team and in different roles, and it’s really nice when we can all learn together so we’re all on the same page, and that was a really big plus and the size was appropriate*. [Learner M] “*I would really like for the nursing staff to have a little bit more of a—some more education about that”.* .” [Allied Health 2] “*Again, we do a lot of undoing of messaging. So, I do feel like having more education—and you’re right, even with nursing and staff, just across everyone, it would help. Because sometimes it does come up, where I have, families will make comments to me like, “Oh, someone told me this,” or, “Someone told me that,” and I’m thinking, that’s never been on the table, but if they’re uncomfortable, they don’t know what else to say.”* [Allied Health 3] “*Well, I think the nursing profession would really benefit all the way from initiating palliative orders. There are a lot of situations where, so say if [palliative care service] is not involved but the doctor has discontinued all the medications, there’s just a lot of PRN medications. So, there are times that, you know, that this is the wish, it’s all charted, and you talk to the family, but it’s just on as needed. So, sometimes they don’t give, because the patient’s just lying there restful, but it doesn’t mean that they’re not in pain, and fully didn’t need it. And then come in to try to turn them and they wake and it’s painful. So, I think education around medication is a big thing of, you know, giving something so that when the next shift comes on and they know that they’re going be turning, positioning, so that the patients aren’t in distress.* [Allied Health 1]
Awareness of Local Resources	27 references across 6 interviews	Increased awareness of resources in the hospital and region, including the local palliative care consultation team. Increased collaboration between hospital and palliative care consultation services.	“T*here is so much about palliative care that is local and specific and so having the combination of the two I think probably made for a real strength, I would guess in the rol out of LEAP here, and in the take up of LEAP, and even, you know, like still all the time referrals are constantly addressed to [provider] and I think family doctors are comfortable texting* [nurse]*, and I expect like a huge part of that is because, you know, they’ve taught the course and they’re very accessible and so everyone still knows them and knows their faces and that; like that really nice local kind of feel, it makes them more approachable.”* [Palliative care Physician B] “*So, letting people know which kinds of resources might be available in the hospital, or even out of the hospital if we’re trying to get people out into the community—beyond the, hey, you can send someone to the hospice or you can do* [home care service]*. Like, for example, one of my patients, the one who had heart failure, at one point, I actually managed to get [home care service] to come in and give her some IV [furosemide].”* [Learner C]

### Symptom management and advanced disease

Improved symptom management, especially in chronic diseases, was described as a major impact by interviewees. This included management of symptoms of patients who were not in the terminal phase of their illnesses, but earlier in their illnesses. In some cases, learners found that the course helped confirm their current practices. The palliative care team described more “upstream” referrals as a major improvement, and that referring physicians and services gave them greater ownership and responsibility for providing a palliative care approach to care.

*“So, initially when before we started the project we were getting a lot of referrals, which were great at our like very low PPS, like 10’s, 20’s, and I think I feel like subjectively I noticed a big difference in that there was like more early upstream referrals that were higher PPS and higher functional status.” (Palliative Care Physician A)*

Learners also appreciated teachings specific to language and communication. Having more difficult conversations, specific to goals of care planning and documentation, had a lasting impact on learners. One hospital staff member recounted a specific interaction with a LEAP learner demonstrating the ability to discuss artificial hydration at the end-of-life, “*… So they’re having, I think, a little more blunt and in depth conversations with people.”* (Allied Health 1)

### Collaborative care

Having colleagues from the same hospital participate in the same session enhanced collaboration. Learning with colleagues from different specialties helped break professional silos across disciplines, and hearing how others may approach a challenging situation was useful. Greater collaboration among course participants and the palliative care team was noted, as was the use of a language related to providing palliative care.

The need to train other staff, including nurses and allied health professionals, was highlighted. This would include knowing how to screen and identify patient’s needs and initiating a palliative care approach earlier, “*I would really like for the nursing staff to have a little bit more of a—some more education about that. So, you know, someone is barely, you know, their level of consciousness is really low, and a full sandwich is sitting in front of them.” (Allied Health 2)*

### Awareness of local resources

Learners appreciated having a preexisting working relationship with course facilitators, as they were familiar with local context and resources and worked in the same hospital and region. Their familiarity with local context and resources was helpful. Both hospital and community organizations have varying policies for referrals, and care provision, making it difficult for hospital-based providers to navigate across varying regions and jurisdictions. Course facilitators were familiar with such policies and how to coordinate and plan care, therefore equipping hospital-based learners on how to plan care effectively. Both learners and hospital staff commented on how policies and referral processes are constantly changing with home and community care, and often struggle with knowing where to go for what.

## Discussion

Across all participant feedback, LEAP Hospital training at a community-based hospital demonstrated hospital-based physicians learning and applying elements of the course specific to increased symptom management of chronic disease, communication through having the appropriate language for discussions about advanced disease and goals of care, practicing more interdisciplinary care collaboration across professional silos, and increased awareness of local resources for navigating local care coordination and discharge planning. Multiple data sources demonstrated recurring themes across participant feedback from post-course evaluations, CTC statements, and interview data. Incorporating multiple data sources provided additional guidelines on recommendations for the “act” phase of this pilot QI project.

The impact of LEAP courseware has been demonstrated in other care settings and modules:^15,17^ this is the first published evaluation of hospitalist-based teaching. Previous investigations of learner experiences also highlighted relevancy to practice and appreciation of increased learner engagement with case-based group learning and open discussion^15^ LEAP has encouraged interprofessional learning, leading to increased networking with colleagues.^17,21^ Peer coaching and the “train the trainer” model of learning, such as LEAP, have been demonstrated to be an effective teaching tool for providing inpatient primary palliative care skills.^22^

LEAP learners identified the need to increase multidisciplinary teaching in future courses. Interdisciplinary learning programs across multiple levels of care organization reported increased knowledge, confidence, and frequency of completing core skills.^21,23^ Furthermore, web-based education modules specific to palliative and end-of-life care have also been demonstrated to facilitate learning and improve knowledge and attitudes for hospital-based providers.^24^ Online LEAP fundamental modules were available to hospitalist learners to complete before “in class” learning, another avenue to help support additional teaching.

Overall, brief learning interventions with hospital-based providers have been successful in spreading the palliative care approach. Learning communication specific to delivering a palliative approach to care for intensivists and critical care staff has led to increased documentation of goals of care within 2 days of ICU referral, decreased medical emergency team calls, reduced aggressiveness of care, and improved symptom management with specific pharmacological therapies.^25–27^

At times, in smaller community-based hospitals, palliative care can be its own subspecialty, with limited understanding of palliative care being a part of everyone’s practice.^28^ Early initiation of palliative care in a community-based hospital setting has reduced hospital length of stay, reduced health care costs, earlier transition to comfort care, and earlier referral to outpatient hospice without having a negative effect on mortality.^29^ Therefore, providing brief educational interventions is one avenue to help increase knowledge and awareness of incorporating a palliative approach to care.

## Limitations

There are several limitations that need to be considered when evaluating this pilot education program. A new hospital electronic medical record system was rolled out corporately during the interviews. That change prevented more allied health and nursing staff from participating in LEAP Hospital education and interviews due to their educational commitments to this system. With the immense human-resource challenges post-COVID-19 pandemic, the hospitalist roster of patients was extremely long; to ensure safe patient care was adequate delivery of care was divided in new ways to meet the increased needs. As a result, care delivery was modified with the duties being occasional or by a locum. Within a 4-month timespan and potentially spending greater than 50% of their clinical day outside the hospital, the providers may or may not have had patients with palliative care needs.

## Conclusions

In conclusion, providing a strong foundation for a palliative care approach was a priority for this hospital site. The LEAP Hospital course was successful in building both knowledge and skills for pain and symptom management and improved communication specific to attending to palliative care delivery. Communication and understanding the appropriate language for having difficult conversations were both learned and applied in practice. Both learner and allied health interview participants described increased interdisciplinary care collaboration and awareness of local resources and navigation for future learning. Future learning initiatives, as a part of the broader quality improvement program, include conducting a local Community of Practice led by the local specialist team where all disciplines can come together to review educational needs and encourage participation in local initiatives. This has been highlighted within the “act” phase of this pilot PDSA cycle and the next iteration of the “plan-do” phase for the education-based QI program. In the future, adding a needs assessment would be beneficial to understand the key areas or topics for discussion to ensure these sessions meet the needs of the participants and thus further inform a PDSA approach for increased capacity building. Furthermore, doing a needs assessment targeting this group specifically (who manage the bulk of hospitalized patients) would be of value to assess whether further educational interventions with this group are warranted.

## Ethics Approval and Consent to Participate

The study was approved by the Hamilton Integrated Research Ethics Board (HiREB # 15478). All study participants provided informed consent.

## Consent for Publication

Informed consent included consent for the utilization of anonymized quotes for publication.

## Data Availability

The data that support the findings of this study are available on request from the corresponding author, A.G. The data are not publicly available due to anonymity of participating facilities and health care providers. To ensure there is no privacy breach, de-identified data can be provided upon request.

## Notes

Supplementary Appendix A1

Supplementary Appendix A2

Supplementary Appendix A3

## Abbreviations Used

CTC: Commitment-to-change
LEAP: Learning Essential Approaches to Palliative Care statements
