# Supplementary material for: “We Can All Learn Together so We’re All on the Same Page”: Impact of a Learning Essential Approaches to Palliative Care Hospital Course on Hospitalists’ Practice
Source: Palliat Med Rep. 2025 May 5;6(1):205–14. doi: 10.1089/pmr.2024.0094 (PMC12410329; doi:10.1089/pmr.2024.0094)
Supplement: Supplementary Appendix A1 [file pmr.2024.0094_supplementary_appendix_a1.docx]

**Appendices**

**Supplement A:** Supplement A: PDSA cycle for Pilot Education QI program

Objective: Test the impact of LEAP Hospital course with a small group of hospital physician staff by assessing their attitudes and comfort with providing a palliative approach to care, and impact on the delivery and coordination of patient care coordination.

1. Plan: Questions include (a) Will learners find the course appropriate for their learning and practice? (b) Will it have an impact on patient care, and care coordination across multiple departments and teams? Using the online course format, a cohort of hospital physicians will participate in LEAP hospital training. Each learner, before and after course participation will compete the LEAP pre- and post-course questionnaires and CTC statements. (Pereira et al., 2020; Pereira, Giddings, et al., 2021). Participants completed a baseline knowledge quiz and rated their attitudes and self-perceived comfort surveys pre- and post-course. Learners and staff who work along the learners but did not participate in the training were invited to participate in semi-structured interviews to better understand course participation and its impact on care delivery.
2. Do: Hospital generalist physicians participated in LEAP hospital training across two sessions, virtually with local course facilitators. Surveys were emailed pre- and post-course participation. Learners and staff were invited via email invitation to participate in semi-structured interviews about their experiences.
3. Study: Twenty-nine physicians completed LEAP Hospital course. Course evaluations demonstrated increased comfort and knowledge when providing a palliative approach to care care. Commitment-to-change statements identified improvements in symptom management, and using the appropriate language to facilitate discussions. Sixteen participants participated in interviews. Learners and hospital staff noted positive impact courses had on symptom and disease management, and enhanced communication when discussing goals of care. Learners enjoyed participating in case-based learning and connecting with peers who are often siloed in practice. Participants also noted the inclusion of allied health for greater collaboration, less didactic teaching, and the inclusion of allied health professionals to ensure all staff are included in training programs.
4. Act: Plan a community of practice for increase discussion-based learning, inclusion of all types of staff, and greater local context on provision of palliative care. Potentially offer additional LEAP hospital training to non-physician staff, and hospital new hires.

HQO Ontario.
