# Supplementary material for: “We Can All Learn Together so We’re All on the Same Page”: Impact of a Learning Essential Approaches to Palliative Care Hospital Course on Hospitalists’ Practice
Source: Palliat Med Rep. 2025 May 5;6(1):205–14. doi: 10.1089/pmr.2024.0094 (PMC12410329; doi:10.1089/pmr.2024.0094)
Supplement: Supplementary Appendix A2 [file pmr.2024.0094_supplementary_appendix_a2.docx]

**Supplement B:** Knowledge Quiz (N = 24 PRE (82%) and N = 14 POST (48%))

|  | **Session A**  January 2022 | **Session B**  February 2022 | **Total**  (Session A + B) |
| --- | --- | --- | --- |
| **PRE**  Score  Average  Percentage  Range | N = 12 | N = 12 | N = 24 |
|  | 132 | 126 | 258 |
|  | 11 | 10.5 | 10.75 |
|  | 55% | 53% | 54% |
|  | 8 - 16 | 7 - 17 | 7 - 17 |
| **POST**  **Score**  Average  Percentage  Range | N = 8 | N = 6 | N = 14 |
|  | 137 | 99 | 236 |
|  | 17.1 | 16.5 | 16.85 |
|  | 85% | 82.5% | 84% |
|  | 14 - 19 | 15 - 18 | 14 - 19 |
| **Score Change**  Average  Percentage |  | | |
|  | + 6.1 | + 6 | + 6.1 |
|  | + 30% | +29.5% | + 30% |
