# Supplementary material for: “We Can All Learn Together so We’re All on the Same Page”: Impact of a Learning Essential Approaches to Palliative Care Hospital Course on Hospitalists’ Practice
Source: Palliat Med Rep. 2025 May 5;6(1):205–14. doi: 10.1089/pmr.2024.0094 (PMC12410329; doi:10.1089/pmr.2024.0094)
Supplement: Supplementary Appendix A3 [file pmr.2024.0094_supplementary_appendix_a3.docx]

**Supplement C:** Commitment-to-Change Statements and Reflections

|  | **Session A**  January 2022 | **Session B**  February 2022 | **Total**  (Session A + B) |
| --- | --- | --- | --- |
| **Initial Commitments**  Number of Learners  Number of Responders  Response Rate  Number of Statements |  | | |
|  | 14 | 15 | 29 |
|  | 12 | 10 | 22 |
|  | 85% | 66% | 75% |
|  | 40 | 48 | 88 |
| **Reflection 4-Months**  Number of Responders  Response Rate Post Course  Response Rate to Learners  Number of Statements |  | | |
|  | 4 | 3 | 7 |
|  | 33% | 30% | 31% |
|  | 28% | 20% | 24% |
|  | 12 | 12 | 24 |
